# SCMR level II/independent practitioner training guidelines for cardiovascular magnetic resonance: integration of a virtual training environment

**DOI:** 10.1186/s12968-021-00836-y

**Published:** 2021-12-27

**Authors:** Amit R. Patel, Sebastian Kelle, Marianna Fontana, Ron Jacob, Jadranka Stojanovska, Jeremy Collins, Hena N. Patel, Marco Francone, Yuchi Han, W. Patricia Bandettini, Chiara Bucciarelli-Ducci, Subha Raman, Gaby Weissman

**Affiliations:** 1 Department of Medicine and Radiology, University of Chicago, Chicago, IL USA; 2 Department of Internal Medicine/Cardiology, German Heart Center Berlin, Berlin, Germany; 3 Division of Medicine, National Amyloidosis Centre, University College London, London, UK; 4 The Heart and Vascular Institute, Lancaster General Health/Penn Medicine, Lancaster, PA USA; 5 New York University, New York City, NY USA; 6 Department of Radiology, Mayo Clinic, Rochester, MN USA; 7 Department of Biomedical Sciences, Humanitas University, Pieve Emanuele, Milan, Italy; 8 Humanitas Research Hospital IRCCS, Rozzano, Milan, Italy; 9 Cardiovascular Division, Department of Medicine, Perelman School of Medicine, University of Pennsylvania, Philadelphia, PA USA; 10 Division of Intramural Research, Cardiology Branch, National Heart, Lung, and Blood Institute, National Institutes of Health, Bethesda, MD USA; 11 Royal Brompton and Harefield Hospitals, Guys’ and St Thomas NHS Hospitals and School of Biomedical Engineering & Imaging Sciences, King’s College London, London, UK; 12 Krannert Institute of Cardiology, Indiana University School of Medicine, Indianapolis, IN USA; 13 Department of Cardiology Medstar Heart and Vascular Institute, Georgetown University, Washington, DC USA

Cardiovascular magnetic resonance (CMR) is an important non-invasive imaging modality used for the evaluation of patients with known or suspected heart disease. Despite its clinical importance, CMR is currently not widely available, in part, because of a scarcity of well-trained physicians to perform and interpret the exam. Moreover, current 2018 Society for Cardiovascular Magnetic Resonance (SCMR) training guidelines [1] and also training guidelines from other societies [2, 3] require a significant amount of in-person hands-on experience making training inaccessible for many individuals. This limits the availability of physicians appropriately trained to perform and interpret CMR exams. The purpose of this statement is to provide guidance for implementing a high-quality virtual CMR training program to complement in-person training options.

By leveraging advancements in video communication platforms, Level II or Independent Practitioner competence in CMR is feasible through the use of a virtual training program that substitutes a portion of the required in-person training with the use of two-way interactive video communication equipment, provided there is active participation between the trainee and the mentor. This two-way communication can be utilized to supplement all elements of a training program, including didactics, protocol selection, image acquisition, and image analysis. A description of the Level II or independent practitioner training requirements is detailed in the 2018 SCMR training guidelines [1].

In order to ensure quality training of CMR is accessible to qualified physicians interested in performing CMR, the SCMR will consider verifying Level II CMR training for those physicians who have demonstrated competence in CMR and have received CMR training as determined by the requirements set forth by SCMR while substituting the in-person, hands-on experience with a high-quality, two-way, video-communication experience. The goal of these recommendations is to maintain high quality CMR level II training and to keep the standards that are currently established for the in-person training environment, while providing guidance for the use of innovative educational approaches, such as the use of virtual training and available digital tools. Acknowledging the importance of the one-on-one interaction between the mentor and the trainee, the interactive sessions of the virtual training (such as the image interpretation and image acquisition) should be limited to a small group of trainees per mentor; whereas didactic sessions could have a larger number of trainees or be pre-recorded.

The image analysis and initial interpretation of a CMR exam remains an essential part of the competency assessment. The percentage of cases that may be replaced by a virtual experience can vary depending on (1) the program logistics and its curriculum, (2) the time spent by the program faculty in mentoring trainees, and (3) the competency of the individual trainee as evaluated by the CMR mentor. Importantly, a final assessment of the trainee’s competency may still require an in-person training component allowing the mentor to observe the trainee’s ability to acquire and interpret a CMR examination.

A high-quality virtual experience should include, but not be limited to, the following stipulations:

- The virtual training program should be fully compliant with the current SCMR training guidelines [1] and ensure the trainee achieves adequate competency to independently perform and interpret CMR studies
- Virtual training program should be led by a Level-III-trained, Advanced Practitioner, or equivalent CMR expert
- Training centers must have a successful track record of on-site training as a pre-requisite for providing virtual SCMR Level II CMR training courses
- Didactic lectures can be provided by faculty selected by the training program. Alternatively, pre-recorded lecture series similar to those provided by the SCMR can be utilized
- In order to allow for direct observation and interaction with the scans as required by the SCMR training guidelines, the trainee must be able to visualize the control console during the CMR scan
- The trainee should have the opportunity to either provide input with sequence selection and modification/troubleshooting based on the clinical question and patient characteristics or have dedicated sessions focused on image planning and trouble-shooting
- The virtual training program should include full interpretation of CMR cases presented in a complete study format with review and feedback from the mentor. These cases can be previously acquired at the training center or obtained through the use of registries such as the SCMR registry to ensure that the full breadth of disease states are encountered by the trainee
- The virtual training program must incorporate safety considerations related to performing CMR. Depending on a trainee’s experience, some components of CMR training such as (but not limited to) stress testing and supervision of individuals with cardiac implantable electronic devices may require an in-person experience
- Video communication equipment should meet local standards for analyzing medical images
- Patient privacy issues should meet local policies and regulations
- The specific remote training plan can be reviewed by SCMR for input if desired by the training program
- The trainee and training program are encouraged to provide feedback to the SCMR about the perceived successes and failures of the virtual training program.

The final determination of a trainee’s competency to independently perform and interpret CMR studies is up to the program director of a training program. This document provides guidance to those developing a virtual CMR training program to complement in-person training. SCMR would be willing to verify that a virtual CMR training program meets the above standards prior to the initiation of the program. While further documents will need to address the future state of CMR training, it is expected that future CMR training will include both a combination of in-person training as well as incorporation of the virtual tools that are available.

Virtual training in CMR provides increased educational flexibility that is currently necessary but its effectiveness will need to be periodically reassessed. The feedback of mentors and trainees will be highly valuable to address future needs in education. The experiences of these virtual training courses will potentially strengthen the ability to train physicians in locations where CMR might not be readily available, and therefore spread its appropriate utilization. Further, adapting a framework that combines virtual and more traditional in-person education may better fit the needs and expectations of newer generations of physicians. We anticipate that future training models will utilize all the tools and techniques available to provide high quality training to a diverse and broad group of physicians allowing for the utilization of CMR in a greater number of locations. The use of new technologies for CMR training will continue to supplement high quality in-person education and lead to a highly skilled and well-trained CMR practitioner workforce.
